# Another umbrella murder? – A rare case of Minamata disease

**DOI:** 10.1007/s12024-020-00247-y

**Published:** 2020-04-22

**Authors:** Anne Albers, Ursula Gies, Hans-Jurgen Raatschen, Michael Klintschar

**Affiliations:** 1 Department of Forensic Medicine, Hannover Medical School, Hannover, Germany; 2 Department of Neuropathology, Medical Centre Bremen-Mitte, Bremen, Germany; 3 Department of Radiology, Hannover Medical School, Hannover, Germany

**Keywords:** Methylmercury, Minamata disease, Umbrella murder, Neuropathological alterations

## Abstract

We report a rare case of fatal intoxication in a 40-year-old man caused by injection of a fluid containing organic mercury, allegedly in an attack with a syringe fixed to the tip of an umbrella. The man suffered from severe neurological symptoms and progressive multiorgan failure and died 10 months later in refractory status epilepticus. Autopsy revealed severe brain atrophy and non-specific kidney damage. Neuropathological examination showed neuronal loss especially in the occipital lobe, distinct granule cell necrosis in the cerebellum and Wallerian degeneration in the brainstem. Postmortem toxicological analysis revealed extremely increased levels of mercury in liver and kidney tissue as well as methylmercury levels in peripheral blood.

## Introduction

In the field of legal medicine, intoxications are challenging cases which often occur without typical symptoms and autopsy findings. Hereby the antemortem as well as the postmortem diagnosis is difficult and often virtually impossible without specific suspicion. Most problematic are homicidal intoxications that are extremely rarely diagnosed (e.g. in New York City there were only three registered cases in a 10 year period), probably not because they are genuinely rare, but because they are severely underdiagnosed postmortem [1, 2].

However, homicidal poisoning is a very popular subject in crime literature and one very prominent scenario that is reportedly used is that of an attack with a modified umbrella (Bulgarian umbrella) that is used to inject a lethal substance [3].

As Hahn et al. reported, intoxications with pharmaceuticals and cleaning agents are most common in Germany but rarely fatal [4]. Another substance which caused numerous individual intoxications as well as public health disasters in Japan and Iraq in the past is mercury [5, 6]. Mercury is a heavy metal which occurs in several forms in the environment and is an essential part of the daily life as component of batteries, thermometers or older dental fillings [7]. Depending upon the application, different mercury compounds are utilized, e.g. in thermometers a mercury thallium compound is used to widen the temperature range [8].

Toxicity of mercury is caused by reactions with the tertiary and quaternary protein structure, based on its affinity to sulfhydryl and selenohydryl groups. As a consequence of this reaction, mercury ingestion affects nearly each structure in the human organism, especially the brain, the renal system and the immunosystem [5]. The target organ is determined by the way of ingestion and chemical form of mercury; while inhaling mercury vapor primarily affects the brain and leads to acute pulmonary dysfunction, mercury salts induce renal failure and gut complications [9]. The most poisonous compounds, however, are organic mercury molecules, most prominently methylmercury, which is converted from atmospheric elementary mercury by water-microorganisms which, in turn, are ingested by fish. The consumption of contaminated fish is the most important source of accidental methylmercury intoxications in humans and caused the Minamata Bay disaster that was due to chronic methylmercury intoxication [5]. Minamata disease might become a problem in upcoming decades because permafrost, which decreases by global warming, stores a globally significant amount of mercury [10]. The actual toxicity of mercury is thus determined by form, dose and rate of mercury ingestion [7]. While large acute exposure causes severe acute inflammatory reactions, chronic low dose ingestion leads to mostly non-specific symptoms [10], and organic mercury compounds like methylmercury lead to damage of the central nervous system. For that reason, patients typically present with visual field constriction, sensory disturbance and cerebellar ataxia. In severe cases methylmercury intoxications can lead to tetraplegia, coma or death. These symptoms correlate with damage of certain regions of the brain; the occipital lobe, cerebellum and brainstem [11].

Nowadays, suicides as well as homicides with mercury compounds are rare [12–15]. Nevertheless, there are several reports of homicidal, suicidal and accidental mercury intoxications in the last centuries. This could be explained by the fact that mercury was used as treatment of different diseases and for that reason easily available [16]. The cases which have occurred in recent years are often associated with victims or perpetrators who are in contact with mercury compounds due to their profession [12]. A well-known example for an accidental mercury intoxication is the case of Karen Wetterhahn (American professor of chemistry) who died from a Dimethylmercury intoxication caused by wearing insufficiently protective gloves [17]. Except for criminal cases and suicides in which metallic mercury compounds were used, there is a particular case report about criminal mercury poisoning using heated tobacco products [13] and one report about a homicidal methylmercury intoxication [15].

Herein we present a rather dubious case of alleged homicide by injection of a fluid containing methylmercury using a Bulgarian umbrella.

## Case presentation

A 40 year old man told his wife that he was pricked with a syringe in his gluteal region without reason or warning by an unknown person while strolling through the city of Hannover. The syringe was fixed to the tip of an umbrella and contained a clear fluid. As he did not have any health complaints, he refrained from contacting the police or a physician. However, after several days to weeks the man showed increasingly reduced general health with fatigue and was finally hospitalized with severe neurological dysfunctions in somnolent status. As his parameters of inflammation were normal, his diastolic blood pressure was high, and his antibody profile and complement activities were normal, sepsis as well as a parainfectious syndrome were considered unlikely. Despite this, his health worsened steadily, and he was treated for what was assumed to be Guillain-Barré syndrome. When the man was tetraplegic, comatose and needed mechanical ventilation, intoxication with heavy metals was considered and a toxicological analysis revealed extremely high peripheral blood levels of methylmercury. A therapy with DMPS was started that led to a gradual reduction of mercury in the blood but finally the man died from a benzodiazepine-resistant status epilepticus 10 months after the incident. A postmortem whole-body CT scan failed to reveal metallic mercury deposits in the body but displayed subluxation of the temporomandibular joint. The clinical course is described in detail by Napp et al. [18].

Police learned of the case after the mercury intoxication was diagnosed, and the investigation revealed a small syringe (typically used for subcutaneous injections, e.g. insulin) containing a fluid with a mercury-thallium bond as well as several beads of metallic mercury bonds at the dashboard (e.g. non-organic mercury, mercury sulphate) of the victim’s car. Furthermore, it turned out that the victim had access to mercury compounds due to his occupation.

### Autopsy findings

An autopsy was performed on the decedent 2 days after death. The height of the deceased was 173 cm and his weight was 69 kg. External examination showed a bite wound in the left corner of the mouth and leg muscle atrophy. The was no injury present on the tongue and the muscles of the neck did not exhibit any injury. Neither of the typical signs of thallium intoxication; Mees’ lines or injection sites or foreign body granulomas at the gluteal region, were found.

The brain, especially the cerebellum, pons and medulla, was atrophic and weighed 1110 g and was without any signs of bleeding or acute ischemia. The right occipital lobe showed a caved area with a diameter of 3 cm and marked cortex atrophy. Both hippocampus regions were of below average size.

The kidneys showed marked edema. In the remaining organs no major pathological changes were observed.

Based on the necropsy findings and the available clinical information the cause of death was ruled to be due to refractory status epilepticus which is compatible with brain atrophy potentially resulting from methylmercury intoxication.

### Toxicological findings

Samples of iliac vein blood (postmortem), liver and kidney tissue as well as peripheral blood (while the clinical course) were toxicologically analyzed. Mercury or methylmercury were found in each sample (Table 1). The blood thallium concentration was <0.2 μg/l (the clinical limit of thallium is <0.6 μg/l).

**Table 1:** Levels of mercury compounds and thallium tested in different compartments. Analysis was performed by gas chromatography – mass spectrometry (GC-MS) and multi-element analysis. Clinical reference values in parenthesis

	Mercury	Methylmercury	Thallium
EDTA Blood (initial measurement)	4255 μg/l (< 2 μg/l)	not tested	< 0,2 μg/l (< 0,6 μg/l)
EDTA Blood (DMPS treatment)	2929 μg/l (< 2 μg/l)	1538 μg/l (< 1 μg/l)	not tested
Venous blood (autopsy)	6,4 μg/l (< 2 μg/l)	3,6 μg/l (< 1 μg/l)	< 0,2 μg/l (< 0,6 μg/l)
Liver (autopsy)	7100 μg/kg (< 490 μg/kg)	not tested	not tested
Kidney (autopsy)	2100 μg/kg (< 9,1 μg/kg)	not tested	not tested

Analysis of the clear liquid inside the syringe which was found in the victim’s car showed that it contained a mixture of mercury and thallium consistent with what is used in low temperature thermometers. These thermometers typically contain 85% or 40% thallium and accordingly 91.5% or 60% mercury, as police reported.

## Neuropathological alterations

**Fig. 1:**
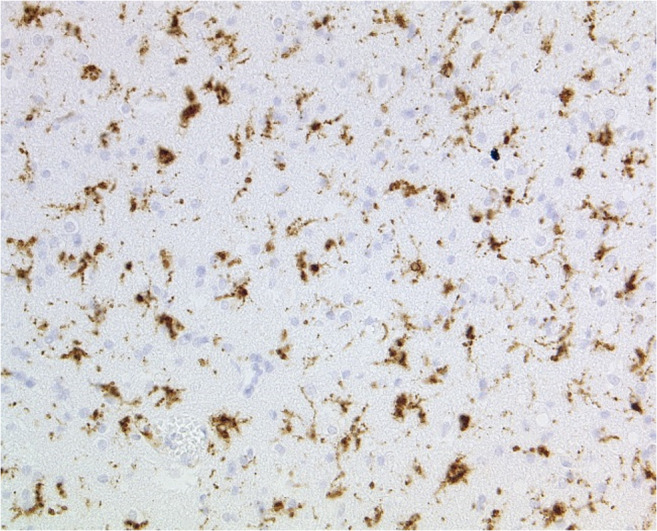
Abundant microglial cells and macrophages in the occipital cerebral white matter (Anti-CD68, original magnification ×100)

**Fig. 2:**
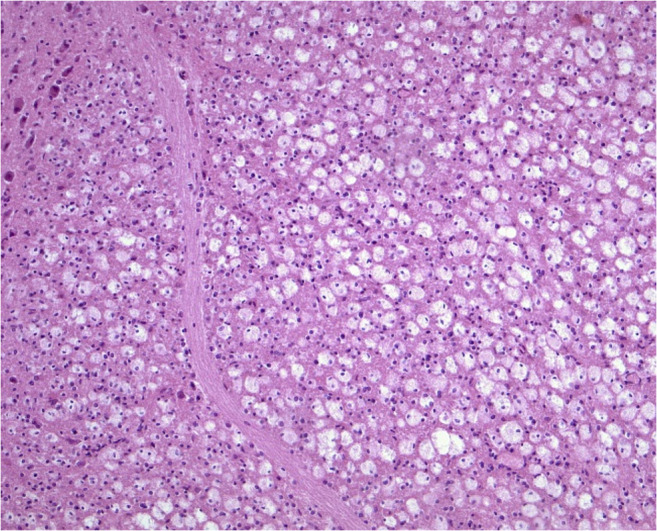
Wallerian degeneration in the pontine tracts with abundant pale macrophages (HE, original magnification ×200)

**Fig. 3:**
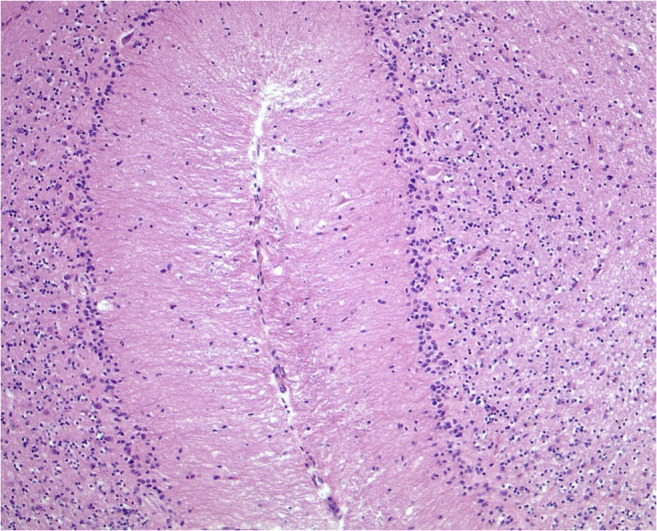
Cerebellar hemisphere with loss of granular cells and Purkinje-cells (HE, original magnification ×200)

**Fig. 4:**
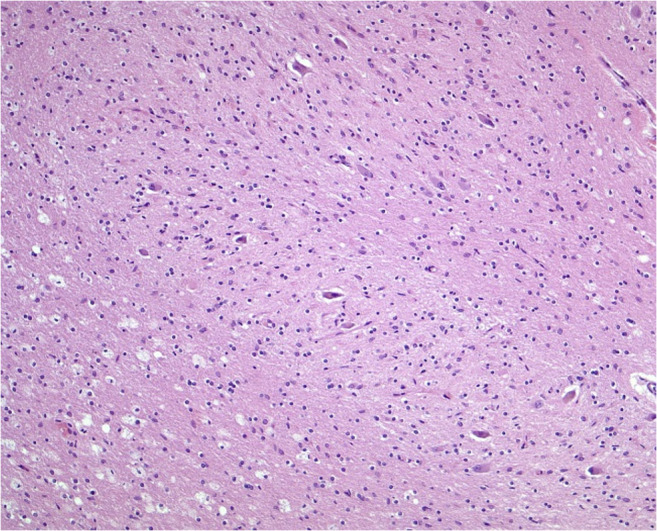
Dentate nucleus with loss of neurons and with macrophages in the white matter (HE, original magnification ×200)

To verify typical mercury associated brain lesions a neuropathological examination was performed. The examined cortex showed focal laminar necrosis and neuronal loss with signs of organization and also a diffuse distributed CD68-positive macrophages and reactive glial cells in the white matter (Fig. 1). The changes were most prominent in the occipital cortex. The caved occipital cortex area presented as an older partial cortex necrosis and a severe form of a laminar necrosis. Wallerian degeneration with many pale macrophages, astrocytes and nearly complete demyelination was observed in the brainstem (in particular in the pons and mesencephalon) (Fig. 2). The cerebellum showed nearly complete granule cell necrosis and degeneration of the Purkinje cell layer with astrocyte accumulations (Fig. 3). Also, alterations of dentate nucleus were characterized by the loss of neurons as well as an increasing appearance of CD68-positive microglia and macrophages (Fig. 4).

Further pathological changes which cause death or are likely to cause death were not found.

## Discussion

Mercury intoxications are especially known from the public health disasters in Minamata and Iraq [5, 6]. There are just a few reports regarding homicides or suicides by mercury compounds [12–14]. The common factor in most cases is that victims or perpetrators of mercury intoxications had access to mercury compounds due to their profession [12].

As Hoppe et al. reported in 2006 the mean blood level of mercury in the general population is 1.7 μg/l [19]. The clinical limit of blood concentration is 2 μg/l of mercury and 1 μg/l of methylmercury. In the present case the blood mercury level was initially increased more than 2000-fold (Table 1). In the postmortem venous blood samples mercury and methylmercury levels were found to be increased 3-fold while there were extremely high mercury concentrations in post-mortem liver and kidney tissue (increased up to 230-fold) (Table 1). The biological effects are, on the one hand, determined by form, dose and rate of ingestion, and on the other hand by interindividual differences in sensitivity to mercury compounds. Mean blood levels of mercury below 10 μg/l are commonly non-poisonous, while extremely increased levels are fatal if left untreated [20]. The clinical course of the patient with initially non-specific neurological symptoms and finally comatose status is expected in cases with such high mercury levels (ca. 2000-fold increased) [11]. Neither specific clinical signs, nor specific (macroscopic) autopsy findings can be expected in such cases. Accordingly, a neuropathological examination and toxicological analysis are indispensable to prove lethal (methyl-) mercury intoxications.

The clinical course of the patient showed a delayed onset of symptoms. This latency is typical for intoxications with organic mercury and can be as long as several weeks or even months [21]. In the tragic and well documented case of Dr. Karen Wetterhahn the symptoms started 3 months after exposition [17].

Neuropathological alterations after methylmercury intoxications are especially known from explorations of Minamata disease [22]. Typical findings, especially in acute and subacute cases, are cerebral edema and ischemic changes with necrosis and neuronal loss due to perfusion disturbances caused by the toxicity. In chronic cases neuronal loss and cerebral gliosis predominate particularly in the pre- and postcentral cortex, the temporal, and the occipital lobes. In consequence, there is a secondary degeneration of associated axons. The cerebellum shows loss of the granule cell layer while there are no pathological changes in the Purkinje layer. This is considered to be a very typical finding in intoxications from organic mercury [23]. Examination of brainstem and spinal cord reveals no primary lesions but Wallerian degeneration [11, 22].

The present case fits best into a subacute form of Minamata disease [22]. Laminar necrosis and neuronal loss dominates the neuropathological observations, but specific distribution with predominate pathological changes in the occipital lobe could also be seen. This was also true for the cerebellum with a loss of granule cells due to the toxicity of mercury also seen in longstanding cases also in addition to a neuronal loss of the dentate nucleus and degeneration of the Purkinje cell layer, most probably caused by disturbance of the perfusion due to mercury toxicity. When considering these results and the absence of other pathological changes and positive toxicological results, the man’s death was caused by methylmercury-related brain damage.

The temporomandibular subluxation displayed by the postmortem whole-body CT scan could be explained by the man’s fatal status epilepticus. It is reported that seizures may, albeit rarely, cause such an injury [24]. The presence of such an injury, much easier to diagnose radiologically than upon autopsy, might thus be used as a sign for death in status epilepticus. In consequence this case further emphasizes the importance of postmortem CT scans.

The police found a syringe in the deceased’s car containing a typical mercury thallium compound. The police investigation revealed that low temperature thermometers at his workplace contained metallic mercury and 8.5% or 40% thallium. Details about possible contaminations with methylmercury were not given by the company. Neither antemortem nor postmortem blood, urine or tissue analysis revealed increased thallium levels. There were also no thallium-specific findings, e.g. Mees’ lines, during the autopsy. In consideration of limited renal elimination and accumulation, when the thallium limit value is exceeded, only a small quantity of thallium could have been injected if the man had used the fluid found in his car [25]. It is therefore not likely that an injection of such a preparation led to the patient’s disease.

Nevertheless, whether the man’s report about the unknown assailant with a prepared umbrella was true could not be unequivocally solved. Neither a motive nor an assailant could be identified. Moreover, the fact that different mercury bonds and an unused syringe containing a mercury preparation were found in the car led the police to conclude that the intoxication was most probably self-administered and not the consequence of an attack by a perpetrator. Therefore, the preliminary investigation was terminated.

Moreover, the story of an attack with an umbrella was very similar to the story of the legendary assassination of the Bulgarian dissident Georgi Markow by a member of the Bulgarian secret service during the communist area, although in that case rizin was used, not mercury [26]. Umbrella murders are also described in several books, films and TV-shows, like one episode of Quincy, M.E., in Navy CIS or the French film Le coup du parapluie from 1980 [3]. Apart from these legends and scripts, there was a series of murders with three separate victims killed using a potassium cyanide filled syringe fixed to an umbrella tip in the south-east of India in 2015 [27].

In summary our case demonstrates typical diagnostic pitfalls of intoxication cases. Therapy was only started after the results of toxicological analysis were received. There were also no specific findings during autopsy, and only extensive neuropathological examinations verified a relation of increased methylmercury levels and death. The lack of specific findings is typical not only for mercury, but for many substances which are not a part of standardized toxicological analyses.

Overall, we think that neuropathological examinations as well as extensive toxicological analyses need to become an integral part of daily forensic routine in cases lacking specific autopsy results.

## Key points

1. (Methyl-) mercury has caused numerous individual intoxications as wells as public health disasters in the past.
2. Organic mercury compounds like methylmercury lead to damage of the central nervous system.
3. There are no specific clinical signs or specific (macroscopic) autopsy findings in (methyl-) mercury intoxications.
4. The neuropathological examination and toxicological analysis are indispensable to prove lethal (methyl-) mercury intoxications.
